# A tribute to James E. “Jim” Heubi, MD

**DOI:** 10.1017/cts.2022.21

**Published:** 2022-02-16

**Authors:** Philip A. Kern

**Affiliations:** Department of Medicine, Center for Clinical and Translational Sciences, University of Kentucky, Lexington, Kentucky, USA

The worlds of research and medicine, and especially the CTSA community, lost a passionate leader with the death of James E Heubi on August 4, 2021. Jim was not only a world leader in bile acid defects and liver disease but also a caring and compassionate Gastroenterology physician who dedicated his career to his patients and their families. As a clinician scientist, he spearheaded clinical research at Cincinnati Children’s Hospital Medical Center and The University of Cincinnati College of Medicine, initially as Director of the institutional General Clinical Research Center (GCRC), and then as the Director of the Clinical Center for Translational Science and Training (CCTST), which contained the NIH CTSA award.

**Figure:**
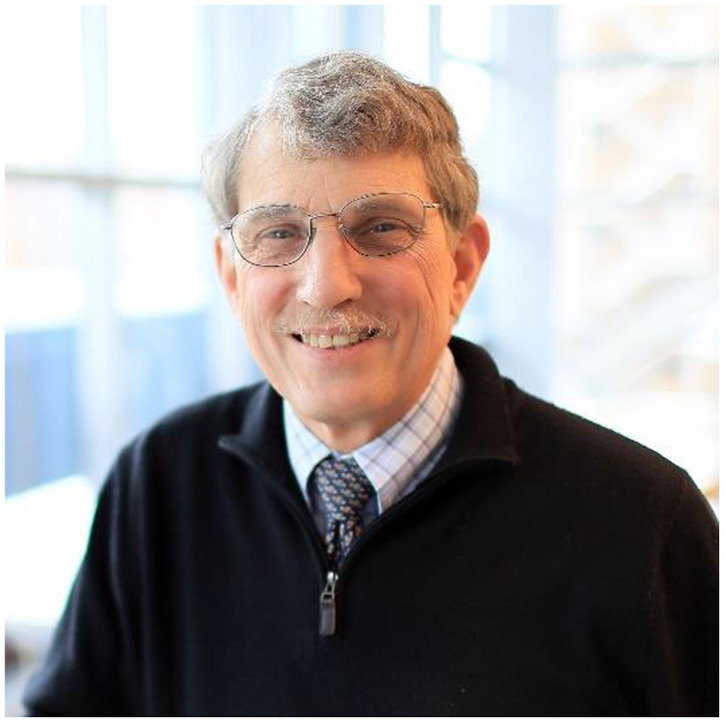

As with the best clinician scientists, Jim was at home in the lab, on the Clinical Research Unit and in the clinic, seeing patients and training the next generation of translational researchers. He brought smiles to the faces of the children he cared for, including many with rare and life-threatening liver disease.

Jim is remembered for his incredible persistence. Back in the mid 70s, an infant was referred to Jim with intractable diarrhea. No treatments were effective and the child was kept alive on TPN. Determined to find a cause for this child’s condition, Jim spent years in research, adopting animal studies to learn how to get jejunal and ileal biopsies. His tissue retrieval allowed him to prove that the defect was the inability to absorb bile acids, which led him down a path over the years to find the mutation responsible. That child, his former patient, is now a fully grown, productive adult.

A leader in the field of bile acid defects, Dr Heubi worked alongside Dr Kenneth Setchell, PhD, to fulfill his dream that patients with orphan diseases affecting bile acid synthesis would have access to life-saving therapies. It was with this vision that they developed a treatment with cholic acid (Cholbam, Travere therapeutics) which was FDA-approved in 2015 and was life saving for children with this rare condition.

Recently, one of Jim’s patients whose life was saved by Cholbam met with Jim’s daughter to show her a letter from Jim stating what an honor and pleasure it was for him to be their doctor. Jim’s family fondly remembers attending the “liver transplant picnics,” Jim would proudly introduce his family to his patients, many of whom were like family to him. He was a tireless champion of pediatric research, inspired by the children he met in his clinical world.

Jim was born in Indianapolis, received his MD from Indiana University School of Medicine, stayed in Indianapolis for his Pediatric Residency at Riley Children’s hospital. Jim was a lifelong Hoosier and an avid IU basketball fan. He came to Cincinnati Children’s Hospital for his GI fellowship in 1975 and never left Cincinnati, rising through the ranks and eventually assuming leadership positions related to his excellence in research, mentoring, teaching, and clinical care. Among his national leadership roles, Jim served as President of the North American Society for Pediatric Gastroenterology, Hepatology, and Nutrition (NASPGHAN).

With a passion for clinical research and an outstanding record of NIH grants and publications, Jim became the Director of the Cincinnati Children’s GCRC in 1988. In 2009, with the GCRC program ending, Jim led the submission of a CTSA grant to fund the new Center for Clinical and Translational Science and Training (CCTST). Jim continued to lead that Center and successfully refunded the CTSA grant multiple times. His vision was that the CCTST could amplify research across the Academic Health Center, bridging pediatric and adult research and dramatically improve outcomes. He sought to improve the way biomedical research was conducted, reduce the time between discoveries and patient treatments, engage communities in clinical research efforts, and train the next generation of clinical and translational scientists. He was tireless in his efforts to harness the resources of the CCTST to improve the health of the community in which it resides. In his own words, “I’m just doing my job. I’m just trying to be as effective a leader as I can, and I really do believe we have to think about the community in terms of how we think about healthcare and how we think about diversity, equity and inclusion – all of these areas are important as we move forward.”

In addition to his research, his patient care and his leadership, Jim is remembered for his innate ability to mentor and encourage all individuals regardless of stage or state in which they find themselves. He has been described as “the mentor’s mentor” and held a genuine confidence in those he guided. He was involved in the career development of innumerable fellows over the years. He always gave willingly of his time and never was one to turn down a request for help. He was very active in summer research programs for students and was honored with the UC College of Medicine Daniel Drake Medal, the highest honor for any faculty member.

Jim will be remembered as a truly passionate physician scientist, who cared deeply for his patients and their well-being, but who was not satisfied with the status quo, and worked from the bench, to clinical research and eventually back to the bedside with a life-saving treatment for disorders of bile acid metabolism. He will be very much missed, not only by his family and his thankful patients, but also by his friends and colleagues who had the pleasure to experience his knowledge, insight, and passionate pursuit of novel treatments. Jim Heubi was a lifeforce that has no comparison.

The following individuals contributed to this tribute: Amy Bunger, Michael Farrell, Erin Haynes, Christine Heubi, Jessica Kahn, Brett Kissela, and Jareen Meinzen-Derr.

